# Brain susceptibility imaging provides valuable *in vivo* insights into cerebellar diseases, but biological interpretations remain elusive

**DOI:** 10.1093/braincomms/fcac007

**Published:** 2022-02-07

**Authors:** Ian H. Harding, Phillip G. D. Ward

**Affiliations:** Department of Neuroscience, Central Clinical School, Monash University, Melbourne, Australia; Monash Biomedical Imaging, Monash University, Melbourne, Australia; Monash Biomedical Imaging, Monash University, Melbourne, Australia; Turner Institute for Brain and Mental Health & School of Psychological Sciences, Monash University, Melbourne, Australia

## Abstract

This scientific commentary relates to: ‘Quantitative susceptibility mapping reveals alterations of dentate nuclei in common types of degenerative cerebellar ataxias’ by Deistung *et al*. (https://doi.org/10.1093/braincomms/fcab306).

**This scientific commentary relates to: ‘Quantitative susceptibility mapping reveals alterations of dentate nuclei in common types of degenerative cerebellar ataxias’ by Deistung *et al*. (https://doi.org/10.1093/braincomms/fcab306).**

The deep cerebellar nuclei are vital to cerebellar function and connectivity with the rest of the central nervous system. Examining and tracking the integrity of these structures are essential, but currently limited aspect of *in vivo* neuroanatomical characterization in most degenerative cerebellar diseases. In this issue of *Brain Communications*, Deistung *et al.*^1^ employ the MRI technique of quantitative susceptibility mapping (QSM) to provide compelling new insights into the anatomy and microstructure of the dentate nuclei across a range of inherited and idiopathic degenerative cerebellar diseases.

The deep cerebellar nuclei are the output hubs of the cerebellum, primarily receiving inputs from the Purkinje cells of the cerebellar cortex, and giving rise to efferent fibres projecting to the cerebral cortex (via thalamus), midbrain, brainstem and spinal cord. In the human brain, the dentate nuclei are by far the largest of the deep cerebellar nuclei and are key nodes in both motor and non-motor cerebello-cerebral circuits.^2^ The post-mortem histological investigations have provided the evidence of direct pathology or reduced synaptic innervation of the dentate nuclei in many degenerative cerebellar diseases.^3^ However, *in vivo* neuroimaging studies of the dentate nuclei in patient populations remain scarce, particularly outside of Friedreich ataxia.^4,5^ Such studies are critical to characterizing the relationship between anatomical changes in the cerebellar nuclei and symptom severity, identifying neuroimaging indicators of disease stage and progression, and where possible, enabling insights into pathological mechanisms.

The scarcity of *in vivo* research reflects the challenges of quantitative characterization of the deep cerebellar nuclei. Their tissue properties render them invisible or poorly defined on conventional MRI images, such as T_1_-weighted or T_2_-weighted acquisitions. Further, while the high iron content of the dentate nuclei does allow them to be visualized using susceptibility-weighted MRI—a technique commonly used clinically for the qualitative detection of vascular abnormalities or haemorrhages—this approach is non-quantitative and prone to artefacts (e.g. blooming). These limitations have largely been overcome through the development of QSM.^6^ QSM allows for more precise mapping of the boundaries of iron-rich brain structures, including the dentate nuclei, alongside empirical study of the susceptibility coefficients themselves. Susceptibility is widely regarded as a proxy for local iron concentration, but as the authors discuss, the non-specific nature of this proxy poses a challenge when interpreting findings.

Deistung *et al.*^1^ utilize QSM to quantify dentate nucleus volume and susceptibility in people with degenerative cerebellar diseases, in many cases providing first-in-disease *in vivo* descriptions of these features. Their work also includes several innovative and important analytical advances. First, they seek to delineate and analyse the outer grey matter shell of the dentate nucleus independently to its central white matter core. This can only be approximated due to the resolution constraints of the acquisition, but moving closer to tissue-specific inferences and respecting the anatomy of the dentate nuclei—which is unique relative to other structurally uniform deep grey matter structures—is critical to providing biologically informed descriptions of disease features.

Second, in addition to deriving the overall measures of dentate nucleus volume and susceptibility, the authors employ a voxel-level analysis approach that allows for regionally targeted inference. This approach reveals the spatial patterns of the effects, allowing the authors to show that anatomical changes in the dentate nuclei are spatially non-uniform and distinct between diseases. The increased richness of the data that are generated also opens the door for potential future applications of more sophisticated or artificial intelligence augmented analytical approaches to, for example, identify 4D markers of disease progression. Such analysis, in parallel with efforts to improve automation of dentate nucleus segmentation, may offer exciting possibilities for translation of QSM to multi-centre and clinical trial contexts.^7^

Finally, the authors analyse group differences in susceptibility both with and without volume correction. Increased susceptibility is observed in many neurodegenerative conditions, and is generally interpreted as a marker of iron accumulation.^8^ However, mean susceptibility would similarly increase if a fixed quantity of iron was instead concentrated within a smaller volume of tissue. Accounting for tissue atrophy when comparing susceptibility values between clinical and control groups putatively allows for the determination of changes in absolute iron levels, as opposed to relative iron concentration. Such an analysis assumes a one-to-one mapping between susceptibility and iron concentration, which as discussed below may not be entirely tenable. But if accurate, such inferences are compelling as they provide for rich descriptions of disease expression and progression, including molecular-level insights, a holy grail of neuroimaging coined ‘*in vivo* histology’.

Taken together, Deistung *et al.*^1^ provide new knowledge regarding the integrity of the deep cerebellar nuclei, and demonstrate the utility of QSM in the neuroimaging toolkit for degenerative cerebellar diseases. But as noted by the authors, determining the biological underpinnings of susceptibility signal changes in neurodegenerative diseases remains challenging. Iron is considered the dominant source of contrast in QSM and susceptibility is well-correlated with iron levels in grey matter structures. But in white matter, susceptibility is also strongly influenced by myelin content.^9^ This is important in the case of the dentate nucleus, where the iron-rich region encompasses not only its grey matter shell, but also the central white matter core of the structure. Loss of myelin, such as reported in the region of the dentate nucleus and adjacent superior cerebellar peduncle in Friedreich ataxia,^10^ would result in increased susceptibility, indistinguishable from the effect of iron accumulation on QSM images.

Different iron species (i.e. free versus ferritin-bound versus haemosiderin-bound) and iron in different oxidative states [i.e. Fe(II) versus Fe(III)] also have different molecular susceptibilities.^9^ Shifts in the relative concentrations of different iron species could therefore lead to susceptibility changes independent to changes in the total iron pool. The influence of other paramagnetic substances in the brain, such as copper, also cannot be fully discounted. Although the concentration of copper in the brain is small compared with iron, and copper would only contribute to the susceptibility signal in its ionic Cu(II) state, shifts in the concentrations of these molecules may also influence the susceptibility signal. The influence of these factors on the susceptibility signal, if any, in degenerative ataxias is currently unknown.

Susceptibility changes in degenerative disorders may therefore be multifactorial, with iron-related alterations a dominant but nonunique contributor. But the biological mechanisms that drive local alterations in iron also remain unclear. As highlighted by Deistung *et al*.,^1^ evidence from other neurological conditions indicate that cell-dependent mechanisms of iron dysregulation, such as mitochondrial dysfunction and impaired cellular metabolism, may operate alongside cell-independent processes including the activation and trafficking of iron-rich microglia to sites of pathology.^11^

Multi-contrast neuroimaging approaches may be helpful in resolving some of these biological uncertainties. Pairing QSM with measures of myelin integrity derived from diffusion or magnetic transfer imaging,^10^ indices of glial activity and neuronal health from magnetic resonance spectroscopy, or markers of neuroinflammation using positron emission tomography may help differentiate the underlying processes.^12^ However, definitively uncovering the biological basis of susceptibility changes in these disorders will ultimately require the pairing of non-invasive imaging approaches with post-mortem histology.

Although interpreting the biophysical basis of susceptibility changes remain challenging, Deistung *et al.*^1^ provide formative evidence towards demonstrating its utility as a biomarker of the disease state. Indeed, multiple pathological processes and epiphenomena may be conspiring together to elicit susceptibility changes in the dentate nuclei, but this may ultimately prove to be a strength rather than a shortcoming of QSM. Their work strongly motivates follow-up longitudinal studies to determine the time course and sensitivity of these measures to disease progression, and indicates that greater focus on the integrity of the deep cerebellar nuclei in degenerative ataxias is essential to establish more complete disease characterizations.

## References

[1] Deistung A, Jäschke D, Draganova R, et al Quantitative susceptibility mapping reveals alterations of dentate nuclei in common types of degenerative cerebellar ataxias. Brain Communications. 2022. 10.1093/braincomms/fcab306 PMC8914888 35291442

[2] Dum RP, Strick PL. An unfolded map of the cerebellar dentate nucleus and its projections to the cerebral cortex. J Neurophysiol. 2003;89(1):634–639. 12522208 10.1152/jn.00626.2002

[3] Koeppen AH . The pathogenesis of spinocerebellar ataxia. Cerebellum. 2005;4(1):62–73. 15895563 10.1080/14734220510007950

[4] Stefanescu MR, Dohnalek M, Maderwald S, et al Structural and functional MRI abnormalities of cerebellar cortex and nuclei in SCA3, SCA6 and Friedreich’s ataxia. Brain. 2015;138(Pt 5):1182–1197. 25818870 10.1093/brain/awv064 PMC5963415

[5] Ward PGD, Harding IH, Close TG, et al Longitudinal evaluation of iron concentration and atrophy in the dentate nuclei in Friedreich ataxia. Mov Disord. 2019;34(3):335–343. 30624809 10.1002/mds.27606

[6] Liu C, Li W, Tong KA, Yeom KW, Kuzminski S. Susceptibility-weighted imaging and quantitative susceptibility mapping in the brain. J Magn Reson Imaging. 2015;42(1):23–41. 25270052 10.1002/jmri.24768 PMC4406874

[7] Schwarz AJ . The use, standardization, and interpretation of brain imaging data in clinical trials of neurodegenerative disorders. Neurotherapeutics. 2021;18(2):686–708. 33846962 10.1007/s13311-021-01027-4 PMC8423963

[8] Ravanfar P, Loi SM, Syeda WT, et al Systematic review: Quantitative susceptibility mapping (QSM) of brain iron profile in neurodegenerative diseases. Front Neurosci. 2021;15:618435. 33679303 10.3389/fnins.2021.618435 PMC7930077

[9] Duyn JH, Schenck J. Contributions to magnetic susceptibility of brain tissue. NMR Biomed. 2017;30(4):e3546. 10.1002/nbm.3546 PMC5131875 27240118

[10] Selvadurai LP, Corben LA, Delatycki MB, et al Multiple mechanisms underpin cerebral and cerebellar white matter deficits in Friedreich ataxia: The IMAGE-FRDA study. Hum Brain Mapp. 2020;41(7):1920–1933. 31904895 10.1002/hbm.24921 PMC7267947

[11] Daglas M, Adlard PA. The involvement of iron in traumatic brain injury and neurodegenerative disease. Front Neurosci. 2018;12:981. 30618597 10.3389/fnins.2018.00981 PMC6306469

[12] Khan W, Corben LA, Bilal H, et al Neuroinflammation in the cerebellum and brainstem in Friedreich ataxia: An [(18)F]-FEMPA PET study. Mov Disord. 2022;37(1):218–224. 34643298 10.1002/mds.28825

